# “We are pests, we have no future”: The prediction of anxiety by perceived discrimination in patients with coronavirus: Mediating role of psychological resilience

**DOI:** 10.3389/fpsyg.2022.979186

**Published:** 2022-11-07

**Authors:** Shuhan Li, Jiayu Gu

**Affiliations:** School of Media and Communication, Shanghai Jiao Tong University, Shanghai, China

**Keywords:** COVID-19, perceived discrimination, psychological resilience, patients with coronavirus, anxiety

## Abstract

In a short amount of time, the COVID-19 pandemic has played havoc on social security, and people infected with coronavirus may have suffered from both physical and mental health issues requiring treatment. The purpose of our study was to examine the effect of perceived discrimination on anxiety in patients with coronavirus and to observe the role of psychological resilience as a mediator in this process. 376 patients with coronavirus were given a questionnaire, and 26 of them participated in in-depth interviews. Our results demonstrated that perceived discrimination in patients with coronavirus was predictive of anxiety and that strong perceptions of discrimination reduced patients’ psychological resilience levels, thereby triggering severe anxiety. Furthermore, psychological resilience was demonstrated to be a significant predictor of anxiety severity. Psychological resilience has been shown to act as a mediator between perceived discrimination and anxiety. As a response to COVID-19, the government, the media, and the general public should treat patients with coronavirus scientifically and rationally, minimize the secondary psychological damage caused by the perception of discrimination to the special groups of society represented by patients with coronavirus during the pandemic, correct the erroneous stigma generated by the traditional communication process, and prevent the spread of the psychosocial virus.

## Introduction

COVID-19 has severely impacted hundreds of nations and regions, including China. The outbreak of the pandemic has posed a threat to public safety and sparked widespread fear, resulting in psychological conflicts like anxiety, sadness, and rage (Li et al., 2020). The explosion of COVID-19 has caused massive damage to the sense of security in society in a short period. Measures to reduce the transmission of the virus have concentrated on prevention, such as social isolation, health blockades, etc. (Jefferson et al., 2020), and although these efforts have been effective, they have also led to psychological issues such as anxiety and stress. The UN advises that “fear, gossip, and stigma” are the greatest obstacles associated with COVID-19 (Un News, 2020).

The number of persons infected with the COVID-19 virus has skyrocketed globally, and discrimination against those afflicted or in close contact with COVID-19-infected individuals has evolved (Lin, 2020). The coronavirus patient group refers to the people with the COVID-19. During the COVID-19 pandemic, Negative public attitudes and beliefs regarding COVID-19 illness, along with a lack of awareness about the Coronavirus and anxieties about its influence on infectiousness and death, have escalated discrimination toward patients with the Coronavirus (Saeed et al., 2020). Public stereotypes include of prejudice, preconceptions, and discriminatory practices, such as denying COVID-1 patients with coronavirus full community participation (Wood et al., 2014). People with unwanted diseases are aware of public stereotypes about their condition, and these stereotypes may lead patients with coronavirus to perceive themselves as members of a stigmatized group, perceived discrimination may lead to a decrease in self-esteem and self-efficacy, trigger negative social comparisons and self-criticism, and provoke anxiety or depression (Boyd Ritsher and Phelan, 2004; Gilbert and Irons, 2008). During the COVID-19 pandemic, patients with coronavirus may have experienced self-panic owing to their perception of prejudice, and it has transformed from a medical sickness to a social “psychological disease”: The long-term effects of COVID-19 will include discrimination in daily life, job, education, and social relationships for many individuals (Tehrani, 2020). Many more will contract COVID-19, and stigma toward COVID-19 survivors can cause anxiety, mental health issues, and social isolation, some patients with coronavirus may conceal their condition for fear of prejudice, and anxiety owing to fear of discrimination can lead to delayed diagnosis, preventing symptomatic individuals from seeking medical care and posing hurdles to COVID-19 management and treatment (Grover et al., 2020). As one of the vulnerable groups, people infected with coronavirus have become a group with more perceptions of discrimination during the COVID-19 pandemic: Perceived Discrimination refers to an individual’s perception of being unfairly treated due to their membership in the group to which they belong (Pascoe and Richman, 2009). According to the learned helplessness theory (Maier, 1976), the perception of discrimination makes the individual feel that the way others treat them is mainly affected by external factors beyond their control. Then negative emotions such as depression and loss occur, eventually leading to depression (Schmitt et al., 2014). As a psychological reality, perceived discrimination strongly impacts vulnerable groups (Dion and Kawakami, 1996; Liu et al., 2020).

Previous research on COVID-19 stigma and discrimination has been predominantly qualitative, and these studies have aimed to combat prejudice against patients with coronavirus through information dissemination (Chopra and Arora, 2020; Singh and Subedi, 2020). It has been argued that the knowledge gained from coping with HIV can be utilized to comprehend and combat the stigma associated with COVID-19 (Logie, 2020). Managing this epidemic demands psychological resilience as well (Barzilay et al., 2020). Psychological resilience is frequently characterized as healthy and adaptable functioning in the face of adversity, and evaluating resilience can lead to therapies for psychological trauma (Southwick et al., 2014). Few quantitative research has examined the relationship between patient psychological resilience and discrimination-induced patient anxiety. There is an immediate need to assess the role of psychological resilience in this patients with coronavirus group, to comprehend the mechanisms that mediate the process by which perceptions of discrimination predict anxiety in patients, and to use this information as a foundation for introducing measures to mitigate the psychological impact of the COVID-19 pandemic.

## Literature review

### Perceived discrimination and anxiety among patients with coronavirus

During the COVID-19 pandemic, the outbreak’s prevention and containment generated dread, anxiety, insecurity, and heightened psychological susceptibility (Haider et al., 2020; Szczesniak et al., 2020); blame and discrimination became people’s attitudes toward patients with coronavirus (An and Catalan-Matamoros, 2020; Dhanani and Franz, 2020). A certain degree of coronavirus phobia emerged during the COVID-19 pandemic, which often triggers xenophobia, discrimination, and prejudice in stigmatizing ways when public health is considered (Asmundson and Taylor, 2020; Noel, 2020). Patients with coronavirus may be discriminated against at the social, they may also internalize and apply discrimination perceptions to themselves, believing that the disease is their responsibility or that they may be excluded, generating self-biased emotions such as feelings of guilt, shame, or anxiety (Albery et al., 2021; Ju et al., 2022). The impact of perceived discrimination is multifaceted, involving stress, mental disorders, anxiety, depression, and substance abuse (Carr and Friedman, 2005; Flores et al., 2008). In the context of the COVID-19 pandemic, not only those currently suffering from COVID-19 but also those who have recovered from the disease also face discrimination (Singh and Subedi, 2020).

Discrimination against patients has occurred in many countries: Spain has experienced severe psychological distress, post-traumatic stress disorder, depressive symptoms, higher levels of stress, anxiety, loneliness, and perceived discrimination during the pandemic in Spain (Fiorillo and Gorwood, 2020). People infected with COVID-19 suffer stigma and discrimination after infection, resulting in socioeconomic hardship, inability to adapt to family, community, and work environments, and adverse effects on physical, mental health, and social well-being; Rising stigma and discrimination against COVID-19 survivors in sub-Saharan Africa are hindering progress in the fight against the pandemic (Peprah and Gyasi, 2020). In Ghana, the pandemic is seen as a mysterious epidemic, a way of atonement for humanity (Cipolletta and Ortu, 2020); COVID-19 survivors and their families face discrimination, exclusion, stigma, and isolation. People avoid patients with coronavirus socially out of fear of being infected, exposing them to prejudiced responses, humiliation, and ridicule from the public. Under this discrimination, COVID-19 survivors may develop post-COVID-19 depression syndrome, anxiety, and psychosocial conflicts (Serafini et al., 2020).

Anxiety is an uncomfortable inner turmoil that emerges when a person is confronted with an imminent and potentially threatening situation, it is a composite emotion made up of tension, fear, rage, and other components, it is frequently accompanied by bodily responses and consists mostly of negative and future-focused emotions (Spielberger, 1966). Anxiety is frequent among patients and healthcare professionals during a pandemic (Labrague and De Los Santos, 2020). Low degrees of worry can drive and generate a sense of personal enthusiasm, but chronic anxiety can be incapacitating and have detrimental physiological and psychological effects on individuals. Numerous studies have highlighted the negative effects of high levels of anxiety, such as loss of appetite, dizziness, sleep disturbances, and physical discomfort, which have a significant impact on people’s social functioning, and higher levels of anxiety have also been linked to impairments in certain physical functions (Lee, 2020). We can see from these facts that prejudice and fear linked with the psychological disorders of the patients with coronavirus have become intractable societal problems and that the suffering of these individuals is a pressing concern as we reach the post-epidemic era.

### Psychological resilience

Psychological resilience refers to an individual’s ability to recover from a stressful incident and has been regarded a protective factor for mental health for a long time, exploration on psychological resilience can be traced back to pioneering experiments on children with schizophrenia conducted in the 1960s and 1970s (Luthar, 2016). Over the subsequent four decades, psychological resilience research has undergone multiple waves (Masten, 2007): the first wave emerged in the 1970s and 1980s and focused on individual psychological resilience and protective factors. The second wave emerged in the 1990s, when researchers increasingly focused on the dynamics of the environment and explained psychological resilience from a developmental systems perspective; the third wave focused on interventions to promote psychological resilience and, in essence, conducted experiments to test psychological resilience theories.

Previous research has demonstrated that psychological resilience may operate as a mediator between bad life experiences and depressive symptoms after a disaster, and that psychological resilience mitigates the deleterious effects of negative life events (Pinquart, 2009). Psychological resilience is the interaction between a person’s protective features and risk factors (in the environment), these protective factors may be external (e.g., family or community support) or internal (the individual’s personality attributes, self-efficacy, temperament, social skills, etc.) (Connor and Zhang, 2006). Individuals with high psychological resilience are able to employ their defensive abilities to “bounce back” quickly in the face of adversity, whereas individuals with low psychological resilience are prone to becoming depressed and overwhelmed (Ruiz, 2015). A person’s psychological resilience may be impacted by adverse life situations, in the presence of an excessive number of negative life events or stress, psychological resilience is vulnerable to sensitization (a reduction in psychological resilience), whereas negative life events and stress within a controlled range can increase psychological resilience and produce a hardening effect. This may be the result of a person’s “self-regulatory mechanisms” being overloaded by an excessive number of stressful events, causing maladjustment and other symptoms (Rutter, 2011).

At the height of the COVID-19 outbreak, when psychological and mental health burdens were at their highest, nurses’ perseverance enabled them to effectively recover (De Brier et al., 2020). Resilience is a crucial buffer that can considerably mitigate the effect of the epidemic on the emotional, psychological, and mental health of nurses (Lorente et al., 2021). Thus, resilience may potentially operate as a mediator to alleviate the emotional difficulties caused by COVID-19-induced discrimination. Despite the fact that several papers have highlighted the potential for heightened stigma and discrimination associated with COVID-19 (da Silva, 2020; Logie and Turan, 2020), few research have evaluated the impact of psychological resilience in the experience of discrimination that leads to anxiety in patients with coronavirus. We employed a questionnaire to study the experiences of COVID-19 survivors, interviews to gain a deeper understanding of how they cope with prejudice, stigma, and social isolation. In this study, utilizing perceived discrimination as the independent variable and psychological resilience as the mediating variable in the “perceived discrimination-anxiety” model, the following hypotheses were developed:

*H1: Patients with coronavirus’ perceived discrimination would be positively correlated with generalized anxiety.*

*H2: Patients with coronavirus’ perceived discrimination would be negatively correlated with patients’ psychological resilience.*

*H3: There would be a negative correlation between patients with coronavirus’ psychological resilience and anxiety.*

*H4: Patients with coronavirus’ psychological resilience mediates the prediction process of perceived discrimination on anxiety.*

## Materials and methods

### Participants

In this study, on May 8, 2022, an in-depth visit was conducted at the Shanghai Mobile Cabin Hospital, which is a centralized admission for patients with coronavirus, and people who suffered from coronavirus were selected as the study subjects using the whole group sampling method on the Questionnaire Star platform, and questionnaires were administered to patients with coronavirus via the sample service function. Before the test, the Discrimination Perception Scale, the Psychological Resilience Scale, and the Anxiety Self-Assessment Scale were utilized as survey tools and the goal, procedure, confidentiality principle, and data usage were discussed in detail to the subjects. The collection of 407 questionnaires occurred on September 30. There were a total of 407 surveys gathered by removing the aberrant questionnaires, such as those with conflicting items and unanswered questions, there were 376 valid questionnaires with a collection rate of 92.4% (Table 1). To investigate the perceived discrimination and anxiety of the patients with coronavirus, we conducted in-depth semi-structured interviews with 26 participants (13 females and 13 men) in their homes or at private restaurants or cafés, with written informed consent from each subject before each session. Before each interview, informed written consent was requested. The interviews focused on the patients with coronavirus’ perceptions of discrimination, Infection experience, and emotional states.

**TABLE 1:** Participants’ statistics.

Variable	Item	Frequency	Percent (%)
Gender	Male	215	57
	Female	161	43
Age	<18	29	8
	18–25	83	22
	26–30	139	37
	31–40	125	33
Education	Less than college degree	71	19
	Bachelor	185	49
	Master	97	26
	Doctor	23	6
Total		376	100

### Measures

All non-trait items in this work were presented with instructions related to “your feelings and thoughts since the COVID-19 outbreak began.” Table 2 provides example descriptors for each question item. Perceived Discrimination, Psychological Resilience, and Anxiety were the three variables in the context of the COVID-19 pandemic.

**TABLE 2:** Reliability test and descriptive for study variable.

Variable		Item	Cronbach α
Perceived discrimination	A1	I feel like I’ve been treated unfairly.	0.95
	A2	I feel like I’m being looked down upon.	
	A3	Compared to others, I feel like I’ve lost some opportunities.	
	A4	People around me in society or life are very unkind to me.	
	A5	I am not welcome in certain public places.	
	A6	People mocked me for being a coronavirus patient.	
	…	…	
Psychological resilience	B1	I can adapt to change	0.91
	B2	I can handle whatever happens	
	B3	Coping with stress makes me feel empowered	
	B4	I can always see the humorous side of things	
	B5	I feel in control of my life	
	B6	I am proud of my accomplishments	
	…	…	
Anxiety	C1	I feel nervous, anxious and uneasy.	0.949
	C2	I’m less productive in my studies/works and unmotivated to do things.	
	C3	It takes me a long time to calm down.	
	C4	It’s hard for me to stop thinking about my coronavirus status and worrying about it.	
	C5	I feel lost, sad and hopeless.	
	…	…	

#### Perceived discrimination

By reviewing previous research, we determined that there are two primary types of discrimination perception measures: self-reported, which examines discrimination perceptions based on individuals’ actual experiences, and attribution framework, which examines discrimination perceptions by determining whether individuals attribute negative events or others’ words and actions to prejudice. This study’s questionnaire was prepared based on previous research and the real circumstances (Postmes and Branscombe, 2002; Krahé et al., 2011), The Discrimination Perception Questionnaire consists of 10 items covering verbal discrimination, avoidance and shunning, and the perception of comparative discrimination, and is rated on a 5-point Likert scale ranging from “strongly disagree” to “strongly agree.” The questions were scored using the Likert 5-point scale, which ranges from “strongly disagree” to “strongly agree” on a scale of 1–5. The higher the score, the greater the degree of discrimination perception; conversely, the lower the score, the lesser the amount of discrimination perception. In this investigation, the scale’s Cronbach α is 0.95.

#### Psychological resilience

In our work, we utilized the Connor-Davidson Psychological Resilience Scale (CD-RISC), which is one of the most popular scales for measuring psychological resilience because it was developed using psychological resilience as a personality attribute and clinical experience. The original scale was created by Connor and Davidson (2003) and consisted of five factors: ability, tolerance of unpleasant emotions, acceptance of change, sense of control, and spirituality. Yu translated and refined itin 2007 (Yu et al., 2007), and the new scale has strong psychometric properties; it consists of 25 items measuring optimism, resilience, and self-improvement. Higher scores indicate greater psychological resilience. The ratings were “never,” “sometimes,” “frequently,” and “nearly constantly.” In this investigation, the scale’s Cronbach α is 0.91.

#### Anxiety

The American Diagnostic and Statistical Manual of Mental Disorders, Second Edition (1968) categorizes the symptoms of teenage anxiety disorders as withdrawal and overanxiousness (Gratz and Roemer, 2004). Based on the previous work, several scientists have concluded that the core of anxiety consists of negative coping (negative cooperation), physical symptoms (nausea, muscular strain, and soreness), negative emotions (worry, wrath, and excessive tension), and negative cognition (false attributions) (Mayer and Salovey, 1997). Anxiety is a sense of unease or dread in anticipation of a potentially dangerous circumstance. Using current standard measures, we evaluate anxiety (Spitzer et al., 2006). Using a simple self-report, the scale can determine if the person is likely to have generalized anxiety disorder. This scale consists of five items with a Cronbach α is 0.949.

### Methods

To investigate the role of psychological resilience in the prediction of perceived discrimination against anxiety in patients with coronavirus, questionnaires and semi-structured interviews were used to gather data, which was then analyzed using spss26.0 and amos26.0. First, using the Harman one-way test, we conducted an unrotated exploratory factor analysis of all variables. We used structural equation modeling to test the role of perceived discrimination on anxiety through psychological resilience, using the variance maximum likelihood method and Bootstrap test for parameter estimation and mediating effects. Does psychological resilience mediate the relationship between perceived discrimination and anxiety? There are several main methods that can be used to test the mediating effect; one is proposed by Baron and Kenny (1986) and is called the causality regression method. And in recent years, many scholars have questioned the method. Other scholars have used simulation studies to evaluate alternative methods (Baron and Kenny, 1986), and studies have shown that more accurate confidence limits can be obtained using resampling methods, and overall, the bias-corrected bootstrap method is the best method. As suggested by Efron and Efron (1979), resampling methods are better and include both non-parametric and parametric bootstrap methods. The most commonly used method is the non-parametric bootstrap method, which uses uniform sampling with replacement. Repeated sampling with replacement is performed under the condition that the probability of each observation before being sampled is equal for each observation point until it is sampled (both 1/n) (Efron and Efron, 1979). In our work, we used a non-parametric bootstrap method.

## Analysis and results

The results indicated that the first common component accounted for 26.27% of the overall variance, which is less than the 40% criteria provided by Podsakoff et al. demonstrating the absence of significant common technique bias variance. We utilized SPSS 26.0 to conduct a bivariate Pearson correlation test between the variables. It was found that perceived discrimination, psychological resilience, and anxiety perception were significantly correlated (*p* < 0.01), where perceived discrimination and psychological resilience were significantly negatively correlated, and patients with coronavirus with strong perceived discrimination had weaker psychological resilience; psychological resilience and anxiety were significantly negatively correlated, and lower levels of optimism, resilience, and self-improvement were associated with higher anxiety. There was a substantial positive association between perceived discrimination and anxiety, with those who perceived discrimination being more likely to experience anxiety (see Table 3).

**TABLE 3:** Bivariate pearson correlation statistics.

*n* = 376	Perceived discrimination	Psychological resilience	Anxiety
Perceived discrimination	1		
Psychological resilience	−0.342*	1	
Anxiety	0.407**	−0.463**	1

*p < 0.05, **p < 0.01.

After testing, the results revealed that the fit indices of the model were χ^2^/df = 1.807, CFI = 0.970, TLI = 0.968, IFI = 0.970, NFI = 0.940, and RMSEA = 0.041, indicating that the model fit was acceptable.

The results of the mediator effect test are shown in Table 4. Perceived discrimination (estimate = 0.069, *p* < 0.001) indirectly influenced anxiety in patients with coronavirus through psychological resilience. At the 95% confidence level. Neither the percentile method for bias correction nor the percentile method for indirect effects contained 0, which implies that the effect was significant (Table 4). Therefore, the mediating effect of H4 psychological resilience between perceived discrimination and anxiety in patients with coronavirus was supported.

**TABLE 4:** The mediating effect of psychological resilience between perceived discrimination and anxiety.

Path	Point estimation	Boot 95% CI		Percentage of total effect	*P-values*
				
		Lower	Upper		(2-tailed)
Perceived discrimination→psychological resilience→anxiety	0.249	Total effect			
		0.179	0.320		0.001**
	0.18***	Direct effect		72%	
		0.111	0.249		0.001**
	0.069***	Indirect effect		28%	
		0.014	0.125		0.001**

***p < 0.001, **p < 0.01, *p < 0.05.

**FIGURE 1:**
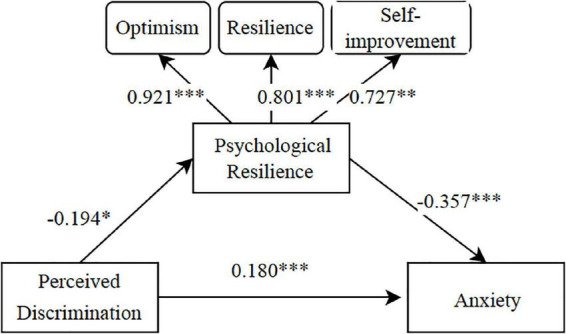
A mediating model of psychological resilience between perceived discrimination and anxiety. ***p < 0.001, **p < 0.01, *p < 0.05.

Figure 1 shows the specific path coefficients, and the provided findings correspond to Table 4. We observe that psychological resilience mediates the relationship between patients with coronavirus’ perceived discrimination and anxiety. Although this indirect effect is not as strong as the direct effect, it is evident that the patients with coronavirus’ perception of discrimination by those around them affects their optimism, resilience, and willingness to self-improvement, and that the reduction of these psychological resilience factors results in increased anxiety. In addition, this result was demonstrated by the semi-structured interviews, some interviewees revealed their experiences and feelings of anxiety in semi-structured interviews: “*Many employers mark in their requirements those who have lived in the cabins hospital, and those who Coronavirus are not wanted. Will I be unable to find a job forever*?” Several respondents indicated that psychological resilience changes also occurred after diagnosis: “*When I returned home after I was cured, only to be verbally abused by my neighbors within the WeChat group (social media) and verbally insulted by my family, I became extremely depressed and concerned about my future.*”

## Conclusion and discussion

During the COVID-19 pandemic, the perceived discrimination was observed to influence psychological resilience and raise the likelihood of anxiety in patients with coronavirus. Based on earlier cross-sectional research demonstrating that discrimination perceptions had a positive predictive influence on anxiety (Kim et al., 2021), our current follow-up study yielded similar conclusions. Additionally, we demonstrated the mediating role of psychological resilience by including it as a mediating variable in the process of the predictive influence of perceived discrimination on anxiety. During the COVID-19 pandemic, stigmatized and discriminated patients with coronavirus identified with particular social stereotypes and prejudices, such as burdens and burdens, and self-managed themselves accordingly. This resulted in a negative self-concept and low self-evaluation, decreased optimism and resilience, and a decreased willingness to improve oneself, which caused low levels of psychological resilience and raised levels of anxiety in patients with coronavirus. Although psychological resilience is merely a view of one’s self-personality and ability and does not necessarily represent one’s actual degree of ability, it can have an impact on a person’s psychological sentiments and emotional state in the face of adversity. People with high levels of psychological resilience have a positive outlook on life, are more likely to think that the challenges they face are passing, are more motivated to better oneself, and see external setbacks and challenges as passing (Southwick et al., 2014). The mediating role revealed that the relationship between “perceived discrimination→psychological resilience→anxiety” accounted for 28% of the overall effect and that, as a relatively stable derogatory perception, perceived discrimination could cause a decline in psychological resilience and an increase in anxiety in patients with coronavirus. In conclusion, all of our presented study hypotheses are supported by the data analysis results. During the COVID-19 pandemic, patients with coronavirus with stronger perceptions of discrimination had lower psychological resilience and higher anxiety, and psychological resilience moderated the primary relationship between perceptions of discrimination and anxiety.

As a widespread uncontrollable and unpredictable pressure, discrimination has a destructive effect on the resources for the protection of an individual’s physical and mental health, reduces self-control, reduces healthy behaviors, and is a risk factor that poses a grave threat to the mental health of patients with coronavirus. Patients discriminated against often see themselves as feared, excluded, and rejected, which is adverse to the fulfillment of predetermined objectives and the acquisition of successful experiences, so diminishing psychological resilience and inducing anxiety. This was demonstrated via our interviews with 26 responders. One recovered person wished to work at an electronics manufacturing, but was denied entry due to a history of COVID-19 infection within 2 months. *“There were no jobs, no housing, and nobody wanted us.”* One interviewee who had been treated and recovered in a cabin hospital said, *“Since I have been healed and released from the hospital for quite some time, why can’t society regard me as a regular person?”* Some of the patients faced prejudice from their relatives in addition to hostility from society. An elderly interviewee with coronavirus and bladder cancer was released from the hospital after his condition stabilized, but his son refused to take him home since he was a virus carrier. Despite having beaten the illness, they were unable to return to regular life since society refused to accept them. *“It was as if the COVID-19 was a permanent mark that could not be removed, separating individuals into two groups; one side was seen as safe and harmless, while the other became a source of danger in many people’s minds.”* The latter are marginalized, and chastised repeatedly in many ways. In the interviews, the respondents said frankly, *“We are pests, and this makes us fearful and apprehensive about the future.”* COVID-19 sufferers are fearful of going outside, interacting with others, and in extreme situations, even touching objects or hugging their children. *“I used to be a really positive person, but the coronavirus devastated me, and people’s harsh attitude toward me made me question if I should return or whether it would have been preferable to die of pneumonia at that time.”* Despite the absence of the infection, their lives continue to be miserable. Unquestionably, it is mentally devastating. *“We cannot deny that some of theis practices are too extreme and narrow-minded and have already caused harm to others, but we must acknowledge pessimistically that behind this discrimination lies people’s powerlessness and helplessness, which leaves me feeling powerless, pessimistic, and anxious about my unfinished schoolwork.”* How to eradicate this broad discrimination produced by the COVID-19, to ensure that the life and work of patients with coronavirus are not regularly impacted by the fact that they had the condition, and to make the epidemic and everyday reach self-consistent is the most pressing issue of the present day.

## Data availability statement

The original contributions presented in this study are included in the article/supplementary material, further inquiries can be directed to the corresponding author.

## Ethics statement

The studies involving human participants were reviewed and approved by the Shanghai Jiao Tong University. Written informed consent to participate in this study was provided by the participants or their legal guardian/next of kin.

## Author contributions

SL: concept and design of the work, acquisition of the data, development of the analysis and interpretation of data, and drafting of the manuscript. JG: concept and the design of the work, acquisition, and interpretation of data, and manuscript revision. Both authors contributed to the article and approved the submitted version.
